# A Task-Centric Cooperative Sensing Scheme for Mobile Crowdsourcing Systems

**DOI:** 10.3390/s16050746

**Published:** 2016-05-23

**Authors:** Ziwei Liu, Xiaoguang Niu, Xu Lin, Ting Huang, Yunlong Wu, Hui Li

**Affiliations:** 1 State Key Laboratory of Software Engineering, Wuhan University, Wuhan 430072, China; lzw@eqhb.gov.cn; 2 Computer School, Wuhan University, Wuhan 430072, China; linxu@whu.edu.cn (X.L.); jieyuth@whu.edu.cn (T.H.); 3 Institute of Seismology, China Earthquake Administration, Wuhan 430071, China; yunlongwu@gmail.com (Y.W.); lihui@eqhb.gov.cn (H.L.)

**Keywords:** mobile crowd sensing, task-centric, participant selection, data integrity, data prediction

## Abstract

In a densely distributed mobile crowdsourcing system, data collected by neighboring participants often exhibit strong spatial correlations. By exploiting this property, one may employ a portion of the users as active participants and set the other users as idling ones without compromising the quality of sensing or the connectivity of the network. In this work, two participant selection questions are considered: (a) how to recruit an optimal number of users as active participants to guarantee that the overall sensing data integrity is kept above a preset threshold; and (b) how to recruit an optimal number of participants with some inaccurate data so that the fairness of selection and resource conservation can be achieved while maintaining sufficient sensing data integrity. For question (a), we propose a novel task-centric approach to explicitly exploit data correlation among participants. This subset selection problem is regarded as a constrained optimization problem and we propose an efficient polynomial time algorithm to solve it. For question (b), we formulate this set partitioning problem as a constrained min-max optimization problem. A solution using an improved version of the polynomial time algorithm is proposed based on (a). We validate these algorithms using a publicly available Intel-Berkeley lab sensing dataset and satisfactory performance is achieved.

## 1. Introduction

With the development of mobile Internet, mobile crowdsourcing systems (MCSs) have become increasingly popular in recent years. The primary notion of a MCS is to select ordinary citizens to collect and share sensory data from their surroundings by using their resource-constrained smart devices for the purpose of task monitoring or addressing a particular problem.

While participating in MCS tasks, users consume their own resources such as battery, physical storage and network flow. It is essential to conserve the smartphone’s resource in order to increase the sensing efficiency and prolong the lifespan of mobile crowd sensing systems. The long-term MCS resource expenses may have a positive correlation to the number of active participants. Therefore, the MCS should use as few active participants as possible. To achieve this aim, a portion of the requested users could be selected as active participants to carry out the sensing task. The residual users are considered as idle ones with low resource consumption until they are selected in subsequent task cycles.

From a data quality perspective, data integrity is likely to be negatively affected when only a part of the users are selected to live-sample the data. Fortunately, when the requested users are densely distributed in the sensing region, it can be generally assumed that the data measurements at neighboring places tend to be well correlated. Therefore, the missing data samples may be reliably predicted based on the actual sensing data submitted by other participants in neighboring regions. Undoubtedly, such a prediction will result in an approximation error due to imperfect correlations or noise. In this paper, we utilize this approximation error to formulate the data integrity loss caused by particular participant selection schemes. The data integrity is better if the approximation error is less. The first question in this paper can be formulated as follows:

*(a)*  How to select an optimal number of selected users as active participants while maintaining the overall sensing data integrity above a preset threshold?

Previously, the spatial correlation was modeled using the sensing coverage region of each sensor. If user A’s coverage region overlaps with the union of the coverage regions of neighboring users, then the user set excluding A can be selected as active participants without affecting the overall sensing coverage, and hence the overall data integrity. As such, participant selection becomes a problem of pruning the minimum number of requested users while maintaining sufficient sensing coverage.

The participant selection problem is similar to the sleep schedule methods of sensors in wireless sensor networks (WSNs). Among the full-coverage-based sleep scheduling methods, LDS is proposed for clustered WSNs [1]. The main idea of the LDS algorithm is quite plain: based on the fact that the RF transmission power between an ordinary node and its cluster head node has a positive correlation to the distance between these two nodes, nodes are selected to sleep according to their relative distance to the cluster head. Hence, nodes farther apart from the cluster head are given higher probability to be put into sleep mode. However, static WSNs are obviously distinct from mobile participatory sensing systems because nodes in mobile participatory sensing systems possess incoercible mobility modes and incentive demands. Recently, having noticed the lack of participant selection methods, several participant-selection schemes have been presented by different researchers. Gaonkar *et al.* proposed a coverage maximization algorithm that records participants’ tracks and selects participants whose availability matches the campaign coverage constraints [2]. Tuncay *et al.* exploited the stability of user behaviors and selected participants based on the fitness of their mobility history profiles [3]. Similarly, most of the existing methods focus on utility maximization or coverage maximization with a constraint budget, but in such user selection methods, participation fairness has not been considered, which indicates a fraction of participants that may complete most of the tasks with a lower price.

In this paper, we propose an explicitly *task-centric* correlation-measuring approach for the spatial correlation between the sample data submitted by participants in neighboring regions/grids. We then make use of this measured correlation to obtain participant selection schemes. The proposed approach in this paper is based on the following argument: *a user in a grid is eligible to set to be idle if its measurements can be recovered by the sensing data of the remaining active participants*.

The participant selection problem can be rephrased as how to identify this subset of requested users that can be set to idle. In this paper, we consider this participant selection issue as a constrained optimization problem: *optimizing the number of active participants subject to the constraint that the performance loss is bounded by a given threshold*.

An exhaustive search for the process of the selection problem may result in non-polynomial time computation complexity and is impractical. Therefore, we propose a novel participant selection scheme algorithm TCPS which possesses a practical polynomial time complexity. The algorithm explicitly measures the approximation error and regards it as a measure to recursively select participants in grids as active ones.

To further reduce the resource consumption and prolong the lifespan of smart devices, we consider the sampling rate of a smart device can be adjusted based on the requirements of different tasks. Thus, to reduce resource consumption of MCS the sampling rate can be lowered in some tasks. Data integrity can be guaranteed by predicting the missing data. However, this may bring in some inaccurate data due to the prediction error. In addition, it is possible that some users may be always selected to participate while others may always be idle. Thus, we consider the second research question as below:

*(b)*  How to select an optimal number of participants with some inaccurate data so that the fairness of participant selection and resource conservation can be achieved, while maintaining adequate sensing data quality?

To maintain the fairness for participants, the MCS organizer will compute the total participation time of all users and the average participation time at the beginning of participant selection, and then queue all users based on the total participation time. Then the organizer will select participants from the front to the end of the queue. If the participation time of selected users exceeds a certain proportion of the average time, they will be put into the end of the queue.

In this paper, this set-partitioning problem is regarded as a constrained min-max optimization problem. We propose an improved version of the polynomial time algorithm based on problem (a). In this algorithm, the error caused by the inaccurate data is separated from the total error and then the sensing data quality threshold is adjusted based on it.

In this paper, preliminary experiments are conducted to evaluate performance of task-centric participant selection schemes and analyze important elements that affect their performance. The remaining of this paper is organized as follows: in Section 2, we discuss related works on the participant selection problem in MCS. In Section 3, we discuss the spatial correlation of neighboring sensing data and formulate the participant selection problem. In Section 4, the task-centric participant selection algorithms are proposed. We report the experimental results in Section 5, as well as analyze some performance comparisons. Conclusions are presented in Section 6.

## 2. Related Works

Some early mobile participatory sensing models do not support multiple tasks so that the participants are only assigned some special tasks [4,5]. Thus, it’s hard to accomplish massive task allocation for early models because many of them lack proper selection methods and randomly choose participants. Since Duan *et al.* firstly presented a participant selection scheme for multiple tasks [6], the incentive mechanism has been taken into consideration in recent multitask-oriented systems [7,8]. In some sense, the collaboration of sensors in wireless sensor networks can be regarded as a selection problem. In [9,10,11], approaches are designed to determine the time needed for activating sensors and the location to place them. However, the mobility of participants and the special incentive demands lead to huge differences between WSNs and participatory sensing.

As for selecting participants, several strategies make good use of participants’ traces. Lu *et al.* concentrated on initiating the process of collecting data in the special area called “bubbles” [12]. In [2], a coverage maximization algorithm is designed to record the tracks of participants and choose some participants with availability to meet the requirements. Similarly, the traces of participants are assumed to be captured ahead of time or only taken into consideration during the occurrence of the sensing activity [3,13,14,15]. However, these approaches depend too much on the participant trajectories, which may result in the privacy leakage. Collaborative sensing is utilized to reduce energy consumption or substitute low-energy sensors by the high-energy sensors in these schemes [16,17,18,19,20,21,22]. Some mechanisms focus on choosing the most valuable participants in the sensing task. A heuristic greedy algorithm is proposed to choose a minimum of participants to fulfill the assigned tasks after quantifying the process of sensing data collection and estimating the collected data scale. This strategy always selects those whose ratio between data value and the incentive cost is maximum with the iterative approach until the budget is exhausted or all tasks are accomplished [23,24]. A geocast mechanism is introduced to accomplish effective task assignment. In this scheme, a greedy heuristic algorithm is designed to select the candidates who can produce the maximizing revenue at every round when the fact that participants are willing to complete the nearby task is taken into consideration [25].

The location prediction technique has been used in the process of task allocation. Offline and online algorithms are presented to solve the participant recruitment problem (PRP) that is how to select the least number of participants required in order to fulfill the given task. The historical data is utilized for the estimation because it’s hard to determine the time and place of participants in real sensing tasks in advance. The algorithm will greedily choose the participants to make the coverage improvement reach a maximum if the current tasks are not finished [26]. Different from previous methods, some systems pay more attention to providing a certain degree of location privacy for participants. A novel two-stage optimization approach is designed to protect the location privacy in the spatial task assignment via utilizing cloaked locations in the global optimization while the precise locations are adopted in a subsequent local optimization [27]. A three-stage recruitment framework is proposed to reduce the privacy risk by decreasing the exposure of participants’ location and context information. In this approach, we focus more on selecting well-suited participants by using geographic and temporal information decided by profiles regarding the transmission and location as well as time [28].

Aiming at identifying suitable tasks to reduce superfluous task expenses, many approaches have been proposed. A green mobile crowd sensing (G-MCS) framework is presented for efficient management so as to minimize the energy overhead after satisfying the sensing task requirements. In the scheme, a quality-driven participant management is proposed to filter the superfluous data by persistently calculating the k-best sensors that should remain active as well as available [29]. Three approaches are designed to optimize the process of task allocation. Firstly, Greedy (GR) using local optimization is proposed to deliver the nearby tasks to the works under the energy consumption constrains. Then the previous approach is improved by giving priority to the places with high location entropy in the Least Location Entropy Priority (LLEP) based on the fact that more tasks take place in the area with more participants. The third approach preferentially takes the tasks with low travel cost into consideration [30]. Riahi *et al.* presented the improving techniques based on utility-driven data acquisition in the situation where more and more data queries come from a mass of different applications. Utility functions are introduced for effective solutions after acquiring query results and cost. The heuristics are utilized to solve the problem of multi-query optimization [31].

## 3. Problem Statements

In this paper, the MCS is assumed to be organized in a cluster topology. In every cluster, to investigate the spatial correlation of data samples, the sensing region is divided into grids of equal size based on the two-dimension coordinate, and users are densely distributed in these grids. A participant selection scheme will be built merely for those grids whose owner cluster consists of *M* grids. A fundamental assumption of the participation selection scheme is that the sensing data sampled by neighboring grids are likely to be correlated. Nevertheless, how to model and exploit such spatial correlation still needs further investigation.

### 3.1. Sensing Range and Spatial Correlation Estimation

**Figure 1:**
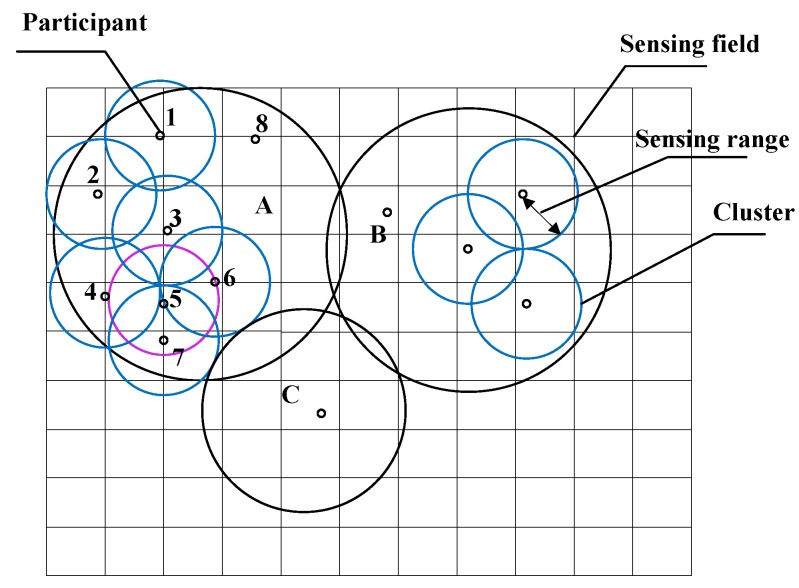
Active participants in a clustered MCS.

For applications such as target detection, an active participant may be assigned a disk-shaped *sensing range*, centered at its location, as shown in Figure 1. If a target presents within an active participant’s sensing range, the participant will detect the target with a pre-specified probability of detection. An active participant becomes *redundant* if its sensing range is fully *covered* by its neighboring active participants. Referring to Figure 1, the sensing range of active participant #5 is completely covered by the union of the sensing ranges of active participant #3, #4, #6, and #7. As such, active participant #5 becomes redundant and can be removed from operation without significantly affecting the overall probability of target detection of the MCS.

For applications such as network monitoring [1], smartphone-based crowdsourcing plays an important role. By subcontracting a monitoring-task to the interested participants, the network-monitoring application can be fulfilled in a participatory manner, using as many participants’ devices as possible distributed in the geographic area of the whole system. Nevertheless, through qualitative analysis, we can draw some interesting considerations. The received signal strength index (RSSI) in the same place may change dramatically with the different wireless communication operators. At this time, how can we select the operator that provides the best quality in those places?

**Figure 2:**
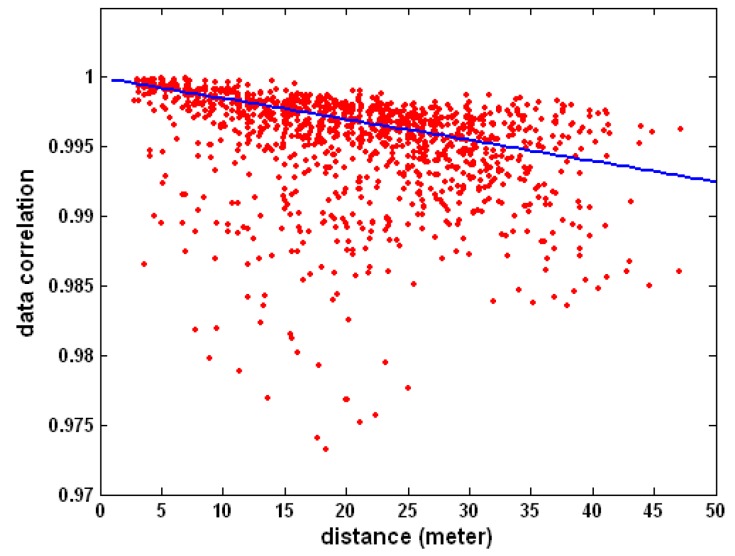
Data correlation *vs.* distance.

To address such a deficiency, we argue that one must use empirical sensing data measurements to directly and explicitly estimate the spatial correlations among neighboring active participants, and to exploit them for the purpose of participants’ idle scheduling. We further assert that the spatial correlation may not even necessarily be decreasing as the distance between a pair of active participants increases. To illustrate this point, we use a publicly available Intel Berkeley Lab sensing dataset [32] which consists of sensing data and location information of all sensor nodes. In Figure 2, we compute the cross-correlation coefficients of the sensor data of each pair of active participants, and plot them against corresponding distance between the active participants. We fit this scatter plot using linear regression and plot the regression line in Figure 2. The line equation is:

(1) *ρ* = −0.00015 × *d* + 1.0 + *δ*

where *ρ* is the spatial correlation coefficient, *d* is the distance (in meter) between active participant pairs, and *δ* is the fitting error which has a normal distribution with zero mean and variance *σ*^2^ = 1.7250 × 10^−5^.

It is clear that although a general trend of reduced spatial correlation with increasing distance exists, the variance is rather significant and there are plenty of outliers. Based on the above observations, we propose a *task-centric* model to represent and exploit spatial correlations for applications where original sensing data are desired. We assume that before normal operation, the MCS will go through a *training* period. During this period, all participants will be active so that the data correlation among spatially separate active participant can be empirically estimated. We further assume that such an estimated spatial correlation will remain unchanged during normal operations in the *testing* period. If deemed necessary, re-training may be performed to update the spatial correlation estimations.

### 3.2. Estimating Sensing Data Using Spatial Correlation

As mentioned earlier, for ecological and environmental monitoring applications, the MCS data will need to be collected and archived for future analysis. When some active participants are turned to idle mode, the data samples that would be measured by these active participants will be lost if the sensing data of these participants are statistically independent on the sensing data of remaining active participants. However, by exploiting spatial correlation among sensing data, it is possible to obtain an estimate of these missing data by using the observed sensing data. Since the correlation may not be perfect, approximation error is likely to occur. We propose to use the expected approximation error per active participant as a criterion to measure the loss of data integrity resulted by putting a portion of active participant s into idle state.

Denote: {***grid_k_***; 1 ≤ *k* ≤ *M*} is the location of these *M* grids {***y_k_*(*t*)**∈***grid_k_***; 1 ≤ *k* ≤ *M*, 0 ≤ *t* ≤ *T* − 1} is the set of participants measurements of ***grid_k_*** during the training period; The selection of grids set in a task time period *I_R_* = {1,2…*R*}; and the all grids set *I* = {1,2,… *M*}.

The set of training sensing data is organized in a *T* × *M* matrix ***Data_M_*** in which the *k^th^* column consists of the *k^th^* grid’s observations over time period [*0*, *T* − 1]. *I_R_* is used to denote a set of *R* (<*M*) indices in {1 ≤ *k* ≤ *M*}. *T* × *R* matrix ***Data****_R_* is obtained from ***Data_M_*** by keeping columns whose indices are in *I_R_*. The remaining columns of ***Data_M_*** then will be denoted as a *T* × (*M − R*) matrix ***Data****_M − R_*. The relationship between ***Data_M_***, ***Data****_R_*, and ***Data****_M − R_* is expressed as:

(2) $$\left[\begin{array}{lll} Data_{R} & ¦ & Data_{M-R} \end{array}\right]=Data_{M}*P$$

where ***P*** is a permutation matrix determined by *I_R_*. *I_R_* corresponds to the *R* active participants. By exploiting the spatial correlation among sensing data, given ***Data****_R_*, one may estimate the missing data ***Data****_M − R_* by projecting it onto the subspace spanned by ***Data****_R_*:

(3) $$D\hat{a}ta_{M-R}=Data_{R}*{\left(Data_{R}^{T}*Data_{R}\right)}^{-1}Data_{R}^{T}*Data_{M-R}=Data_{R}*B$$

where the *R* × (*M − R*) matrix:

(4) $$B={\left(Data_{R}^{T}*Data_{R}\right)}^{-1}Data_{R}^{T}*Data_{M-R}$$

which is the spatial correlation matrix between data measured at the active participants (*I_R_*) and data measured at remaining active participants. Thus, for any *R* × (*M − R*) matrix **D**:

(5) $${\left\|Data_{M-R}-D\hat{a}ta_{M-R}\right\|}_{F}^{}={\left\|Data_{M-R}-Data_{R}*B\right\|}_{F}^{}\leq {\left\|Data_{M-R}-Data_{R}*D\right\|}_{F}^{}$$

where ‖***Data***‖*_F_* indicates that the Frobenius norm be the same with the square root of the sum of squares of all elements of ***Data***. However, from Equation (4), it is clear that to compute the **B** matrix, ***Data****_M − R_* must be known. During the training period where all sensing data are known, the **B** matrix may be estimated. An important assumption of the data centric approach is that this estimated spatial correlation matrix **B** shall remain unchanged in later normal operations when the corresponding ***Data****_M − R_* matrix is unavailable. For convenience, let us denote ***Data*′***_R_* to be the sensing measurements corresponding to grid indices *I_R_* during normal operation; and ${\hat{Y}}_{K-R}^{'}$ to be the estimated measurements corresponding to those idle participants using ***Data*′***_R_*. Since ***Data*′***_M − R_* is unavailable, our approach is to use the **B** matrix obtained using the training data to estimate$D\hat{a}t{a^{\prime }}_{M-R}$:

(6) $$D\hat{a}t{a^{\prime }}_{M-R}=Dat{a^{\prime }}_{R}*B$$

The implicit assumption we make here is that the spatial correlations estimated during the training period would remain the same during the subsequent normal operation. To account for slow time variation of the spatial correlation, retraining of the MCS may be performed periodically at pre-scheduled intervals.

### 3.3. Data Integrity Metric

Different participant selection schemes lead to different choices of active participant index set *I_R_*. To determine which choice is better, a criterion is needed. With a *task centric* approach, we argue that a better choice of *I_R_* should be one that yields smaller approximation error as defined in Equation (5). To account for different size of the active index set *R*, in this paper, we propose to use a relative approximation error as a performance metric to compare different choices of *I_R_*:

(7) $$C=\frac{{\left\|Data_{M-R}-D\hat{a}ta_{M-R}\right\|}_{F}^{}}{{\left\|Data_{M-R}\right\|}_{F}^{}}$$

The purpose of the MCS organizer is to determine the active participant index set *I_R_* to minimize *C*. Note that 0 ≤ *C* ≤ 1. Strictly speaking, *C* should be called a metric for the *loss of data integrity* as it measures the loss of sensing data fidelity due to participant selection. Also note that this cost function is useful only during the training period when ***Data****_M **−** R_* is available. But it is sufficient since in a task centric approach, the set of active participant are determined during training period using training data measurements.

## 4. Task-Centric Cooperative Sensing Scheme

In this section, we discuss the task-centric cooperative sensing scheme to select participants for mobile crowdsourcing systems. In order to save resource consumption, the scheme is to select some participants from all requested users subject to certain information integrity constraint and then set others to idle mode. Considering the sensing workload unbalance problem, we develop the participant selection scheme.

### 4.1. Participant Selection Scheme

Given a set of active participant indices *I_R_*, in Section 2, an optimal linear estimate of the missing sensing data is proposed in Equation (6). The optimality is conditioned upon the assumption that the spatial correlations remain unchanged during normal operation. A performance metric is also proposed in Equation (7) to compare the loss of data integrity due to different choices of *I_R_*. In this section, we will focus on the question of choosing specific composition of *I_R_*. The following is the research problem that we consider in this paper:

Given M users in a MCS, and an upper bound of allowable loss of sensing data integrity loss_threshold (>0), find a participant selection algorithm that minimize the number of active participants R subject to C ≤ loss_threshold.

Selecting *R* active participants out of total *M* potential participated users require exhaustive evaluation of M!/[R! (M-R)!] different combinations. This computation complexity grows exponentially with respect to the size of *M.* Thus direct enumeration of all possible solutions is not practical.

We present a greedy heuristic participant selection algorithm to select the minimum set of active participants in an iterative manner, namely *Task Centric Participant Selection* (TCPS) algorithm. In TCPS, it is assumed that all grid-participants (only one participant is selected to represent the grid) are selected as active participants initially. In each iteration, the active participant list is exhaustively examined to identify exactly one participant to be removed from this list. This removed participant is chosen such that its removal will cost least increase in the loss of sensing data integrity value *C*. The recursion terminates when *C* exceeds a pre-defined upper bound *ε*. This is a greedy heuristic because once a user is chosen to be set to idle mode; it will not be restored in the later iterations. As such the computation complexity is proportional to a polynomial O(*M^2^*). The pseudo code listing of the TCPS algorithm is as shown in Algorithm 1.

Before participant selection, all users will remain active during the training period so that both ***Data****_R_* and ***Data****_M − R_* are available in order to estimate the correlation matrix **B** and the set of active participant indices *I_R_*. Such computation can take place at the cluster head. Once the remaining selected *M − R* users are set to idle, the MCS system will operate with *R* participants, and produces.

**Algorithm 1:** Task Centric Participant Selection (TCPS).**Ensure**  grids Index set *I* ← {1, 2, …, *M*}  iteration count *r* ← 0  Data ← Data*_M_*,  Y ← empty matrix  *selection set of grids I_R_* ← {1, 2, …, *M*}  *data integrity loss_threshold*  *flag* ← false**while**
*Flag = False*  *r = r +* 1,  Selected_id ← 0  *ε_min_* ← *∞*  **for**
*k =* 1 to *M − r +* 1,    X ← Data matrix with the *k^th^* column removed    Y_tmp_ ← [Y | *k^th^* column of Data matrix]    $\varepsilon \left(k\right)\;\leftarrow \;\frac{{\left\|Y_{tmp}-X{\left(X_{}^{T}X\right)}^{-1}X_{}^{T}*Y_{tmp}\right\|}_{F}}{{\left\|Y_{tmp}\right\|}_{F}}$    **if** ε(*k*) < *ε_min_*, **then**     *ε_min_* ← *ε*(*k*)    k* ← *k*    **end if**  **end for**  *Data = Data* matrix without the k* column  ***if***
*ε_min_ > loss_threshold **then***     *Flag = True*  ***else***     *Flag = False ;Selected_id* ← k*  ***end if***  Y = [Y | k* column of Data matrix]  *I_R_* ← *I_R_* without Selected_id k*  *C*(*r*) = *ε_min_***end while****Output**:selection set of grids ← I_R_$Data_{M-R}=Y,\;Data_{R}=Data$$B={\left(Data_{R}^{T}*Data_{R}\right)}^{-1}Data_{R}^{T}*Data_{M-R}$

***Data*′***_R_* as the active participant’s data samples. The idle users’ data samples ***Data*′***_M − R_* can be estimated by multiplying the matrix **B** by ***Data*′***_R_*.

#### Discussions

Question (a) is expressed as a bound optimization problem where the aim is to use the optimal number of active participants to achieve the desired performance in terms of data integrity. Neverthless, it alone does not explicitly address the problem of extending life-span of MCS.

In MCS systems, the sensing data of some regions changes slowly. In such tasks, participants do not have to sense data with high frequency. The sample of participants’ smart device should be adjustable based on the particular requirements of tasks.

Another potential shortcoming of the TCPS algorithm is that the workloads of individual users may not be balanced. Some users may never be selected throughout the entire MCS task cycle, resulting in unfairness.

### 4.2. Improved Participant Selection Scheme

In this section, based on the *TCPS* algorithm, we explore an improved participant selection scheme called IPSA in the MCS system. In consideration of the fairness and consumption, two key factors of the MCS, we can make the system work more efficiently. Sorting the residual power of all participants in every task cycle and dynamically choosing the high-power participants to fulfill the tasks can guarantee that every participant can take part equally in the sensing task.

Meanwhile, by making the full use of current sample data which changes slowly and can be predicted in the next time we can further cut down the counts of our participant selection set, thus reducing the power consumption of the MCS. However using the predicting data will result in inaccurate data and make the selection more unreliable, so we propose a solution coping with the extra error.

Initially all sensing participants are assigned to be chosen from the MCS system into the active set, and we set a priority queue to sort the sensing ability of participants over the energy consumption for the sensing tasks. Then we set a threshold of these participants’ energy consumption on the sensing tasks to make the sensing participants which have worked for a long time to be set idle in the next task cycle. In this algorithm, the span of the MCS system can be extended *N* times where *N* is the task frequency number because the algorithm select a set in every task cycle instead of only selecting for one time.

As defined in the second part of this paper, the matrix of real sensing data is defined as:

(8) $$\;Data_{R}=\;\left[grid_{K_{1}},grid_{K_{2}},\ldots ,grid_{K_{R}}\right]$$

With some inaccurate sensing data as substitute for real sensing data, such as:

(9) $$\;Dat{a^{\prime }}_{R}=\left[grid_{K_{1}},grid_{K_{2}}{}^{\prime },\ldots ,grid_{K_{R}}\right]$$

In the matrix *Data’_R_*, *grid_k2_’* is an inaccurate data. Then we use the equation to calculate matrix *B*:

(10) $$B={\left(Data_{R}^{T}*Data_{R}\right)}^{-1}*Data_{R}^{T}*Data_{M-R}$$

To judge the error from this inaccurate data from the loss of data integrity, we define a compensation matrix to correct the *Data_R_*.

(11) $$\Delta E=[0,\Delta E_{2},0\ldots \;0]\;$$

where *∆E_2_* can be obtained from the observation offset errors proposed in those existing spatio-temporal correlation-based data collecting works [33].

To indicate the inaccurate data, the true data matrix *Data’_R_* can be obtained from the compensation matrix and the actual sensing data matrix *Data_R_*:

(12) $$Dat{a^{\prime }}_{R}=Data_{R}+\Delta E$$

Then if we use this inaccurate data *Data_R_* to estimate the data of idle sensing regions *Data_M −_*_R_ by Equation (6):

(13) $$D\hat{a}t{a^{\prime }}_{M-R}=Dat{a^{\prime }}_{R}*B$$

Then the errors caused by *∆E* we define as follows:

(14) σ(k)=ΔE*B,k∈1,2,...,M−R

where *∆E* is the error value matrix as a result of selecting sensing region K_2_’, we define:

(15) $$\Delta C=max\left\{\frac{\sigma \left(k\right)}{{\text{Data}}_{M-R}}|k\in 1,2,...,\;M-R\right\}$$

To judge the error caused by inaccurate data from the data loss for selecting *I_R_*, we need to correct the formula, we define the new threshold of data integrity loss:

(16) loss_threshold’ = loss_threshold − ∆C

Based on this, in a time period we can select a set *I_R_* if the follow formula is correct:

(17) $$C=\left|\frac{Data_{M-R}^{'}-D\hat{a}ta_{M-R}}{Data_{M-R}}\right|<loss\_threshold'$$

The IPSA is described in detail in Algorithm 2.

**Algorithm 2:** Improved Participant Selection Algorithm (IPSA).***Input:****Training data **Data**.**I_s_ is the finally set of active participant selection.****I_A_*** ← ***I****, **I_s_*** ← {1, 2, …, *M*}*ε_min_*
 ← *∞, **Y**_A_*
 ← ***Data_M_, Y**_B_*
 ← 
*∅,*
*iteration count r*
 ← *0**priority queue A =* [1,2,...,M]***for time_periods_count***
*1 **to** N* ***for***
*i* ← *1 **to** M*  *r*
 ← 
*r + 1*  *average_grid_value*
$\;\leftarrow \;\sum_{1\leq j\leq M}y_{j}(t)/M$ ***for***
*j* ← *1 **to** M*  ***if***
$\frac{average\_grid\_value-y_{j}(t)}{average\_grid\_value}>\;0.2$*queue_sort (M, j)*
 /*make a sort of residual power */ ***for***
*k = 1 to M − r + 1,*   *X*  ←  *Y_A_ matrix with the data set of I_A_*   *Y_tmp_* ← *[Y _B_| k^th^ column of Data matrix]*   $\varepsilon \left(k\right)\;\leftarrow \;\frac{{\left\|Y_{tmp}-X{\left(X_{}^{T}X\right)}^{-1}X_{}^{T}*Y_{tmp}\right\|}_{F}}{{\left\|Y_{tmp}\right\|}_{F}}$   ***if** ε(k) < ε_min_, **then***   *ε_min_*
 ← *ε(k), k* ← k* Data = Data matrix without the k* column ***if***
$\varepsilon_{min}\;>\;loss\_threshold^{\prime }$***then***   *Flag = True* ***else***   *Flag = False*;   *Selected_id* ← k* ***end if*** *Y =* [ Y | k* column of Data matrix ] *I_s_* ← I_s_ without selected_id k* *C(r) = ε_min_****end***
**Output**: selection set of least grids ← *I_s_*

#### Discussions

As we have known, one potential shortcoming of the TCPS algorithm is that the workload of individual users may not be balanced. Some users may never be selected throughout the entire MCS task cycle, thus resulting in unfairness. This problem has been handled in the IPSA, which sets a priority queue and continuously modifies the queue in every task cycle based on the residual power of the participants. All the participants have a chance to be selected to be set active mode in a task in this solution for the reason that if a participant has worked for a long time then its priority will be less than that of other participants which have never been selected. In such a selection strategy based on priority queues, we can always select the high-power participants in a certain task cycle to make sure every participant can join in the tasks.

For another problem of resource consumption, representing a full utilization of resources, the IPSA takes some inaccurate sample data as a substitute for real sensing data and then uses the TCPS to find an optimal participant selection set among the other participants. In this way, we can further reduce the number of participants and save more power because we have gained some data for foundation. Although using these special sensing regions and inaccurate data will result in extra errors, we have proposed a solution to handle the offset errors and ensure that our selection strategy is based on sufficient data integrity.

## 5. Performance Evaluation

In this section, experiments using real-life environmental data (sensor network data set in Intel-Berkeley Lab [32]) are performed to: (i) evaluate the properties; (ii) prove the feasibility; and (iii) compare the performance of the proposed algorithm. All experiments use the following three measurement metrics:

- *Number of selected active participants*: the number of selected active participants generated by the algorithm for the whole task set in each sensing interval.
- *Number of participated tasks*: the number of participated tasks for each participant for the whole task set over the sensing period.
- *Approximation error*: the expected approximation error per participant which is a criterion to measure the loss of data integrity caused by putting a portion of the participants into idle mode.

**Figure 3:**
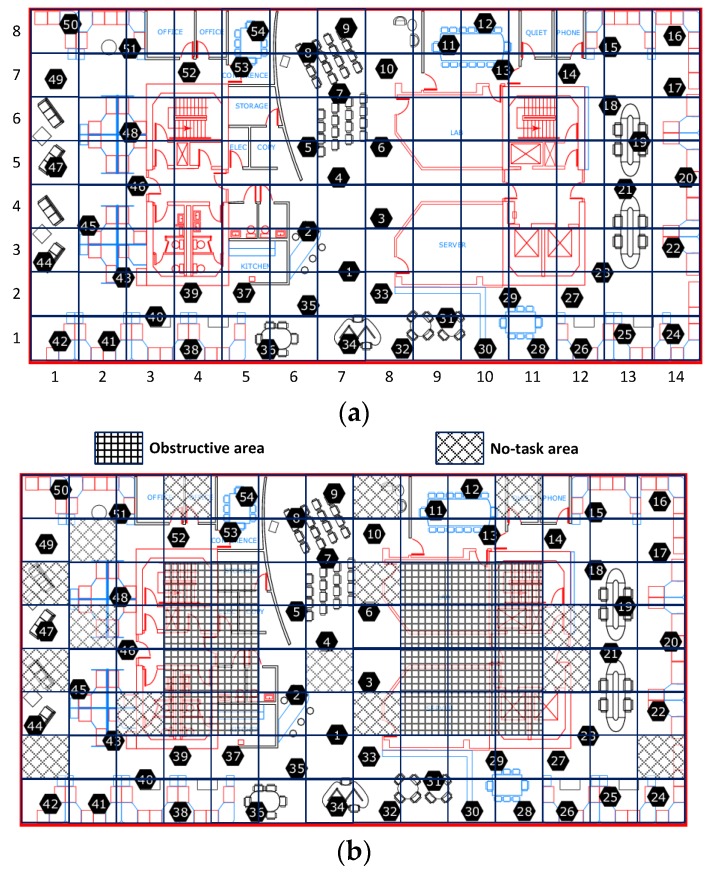
Simulation setup and environmental observations in Intel Berkeley Research lab. (**a**) Sensor deployment and grid-based task area; (**b**) Possible task areas and participant-moving areas.

All results reported here are the averages from multiple runs over different periods from the Intel-Berkeley Research Lab data set. To simulate cooperative sensing in mobile crowd-sourcing systems, we use real life environmental monitoring data from the Intel-Berkeley Lab sensing dataset, in which the sensing samples are humidity and temperature readings taken from 54 wireless sensor nodes between 28 February and 5 April 2004. The sampling interval is 31 s per sample. A layout of these sensors is shown in Figure 3a. The reason we pick the Intel-Berkeley Lab sensing data set is that it is the only environmental data available to us which has fine-grained geographically-distributed and temporally consecutive environmental observations. As shown in Figure 3a, the whole sensing field in the Intel-Berkeley Lab is divided into 112 (14 × 8) grids of equal size served as the sensing task unit areas. The length and the width of the whole sensing field are 41 m and 32 m respectively. Since some grids are missing sensor nodes, they are treated as obstructive areas and no-task areas which are excluded from this experiment, as shown in Figure 3b. Specifically, there is no task releasing in either obstructive areas or the no-task areas, or participant movement in the obstructive areas. In this simulation experiment, there are 100 participants moving in the Intel-Berkeley Lab. All the participants are randomly placed within the whole area. The random waypoint [34] model is selected as the mobility model, with varying maximum mobility speeds (*i.e.*, 0.25, 0.5, 0.75, 1 m/s), and the same pause time of 900 s. We use 11 days of data in which there are few sensor nodes suffering missing data records from the dataset to perform various experiments. In this simulation experiment, we only choose subsets of grid sectors as working-grids and subsets of corresponding participants in the working-grids as active participants in our participatory system for every sensing task interval (*i.e.*, 31 s). The selected active participants will sense data around their grids during the assigned time interval. We use the 11-fold cross validation to examine the approximation errors of the 11 days’ worth of Intel-Berkeley Lab sensing data. Specifically, we treat one day as the testing time unit. Then, one day’s data from the 11 days’ data is selected in turn as the testing data and the remaining 10 days as training data. During the testing period, if a participant is selected as being put into idle mode, its data readings then will only be used to compute the approximation error for the purposes of performance comparison.

### 5.1. Determining Number of Active Participants

**Figure 4:**
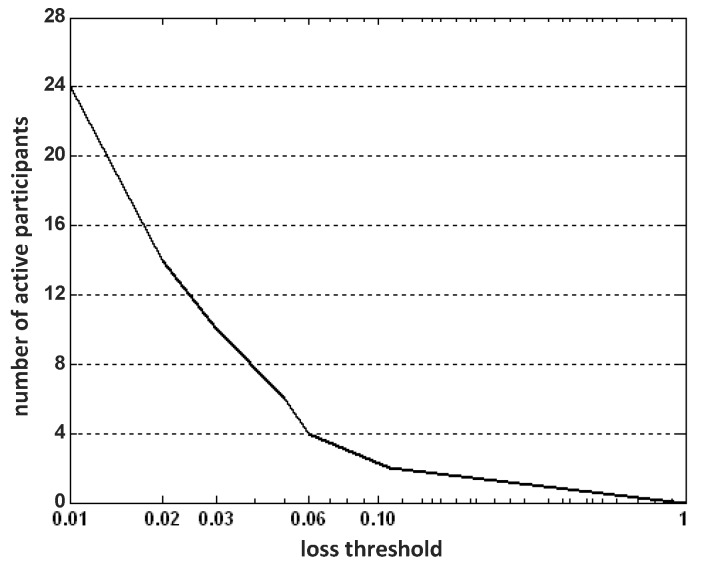
Number of active participants *vs.* loss threshold.

In IPSA, the number of active participants/grids is determined according to the loss threshold. The goal is to use optimal number of active participants under the constraint that the approximation error (of the training data set) is kept below the threshold. In Figure 4, we plot the participant numbers *versus* the loss threshold (over the range of 0 and 1) running the IPSA algorithm.

As expected, along with the increase of loss threshold, the required optimal number of active participants decreases. More specifically, when the loss threshold exceeds 20%, only one active participant is needed. This shows that the data same collected from the neighboring region is highly spatially-correlated. On the other hand, it is worth noting that only 24 participants will be sufficient to estimate the remaining idle participants’ measurements within 1% error.

**Figure 5:**
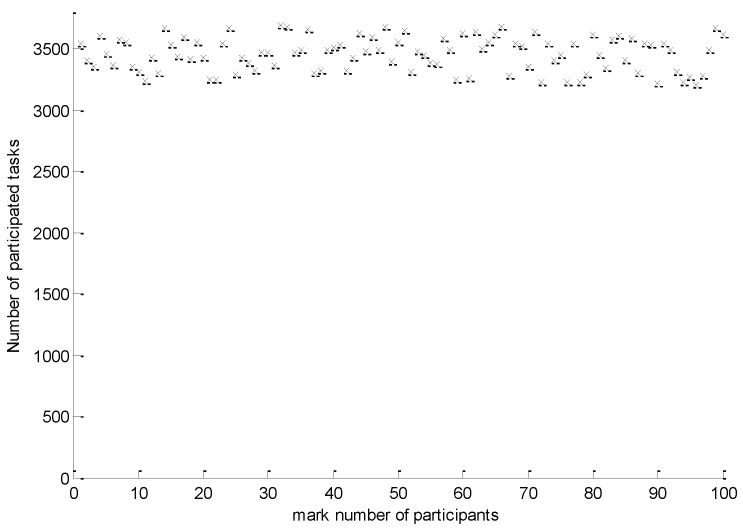
Number of participated tasks for each participant within 8 days.

As shown in Figure 5, the number of participated tasks for each participant for the whole task set over the 11 days’ testing sensing period ranged from 3205 to 3685. Each participant performed an average of 3456 tasks. The standard deviation of the participated tasks for all participants is 136.7, which confirmed that the IPSA can maintain good fairness among participants.

### 5.2. Effects of Variations of Spatial Correlations

A key assumption made by the TCPS and IPSA algorithms is that the spatial correlation matrix **B** estimated using training data will remain unchanged during normal operation (testing data). If this assumption is correct, then the approximation error computed using training data should have similar magnitudes as the approximation error computed using testing data.

To test this theory, we plot approximation error for each day of training and testing period. The day-by-day approximation error is calculated using the formula:

(18) $$\varepsilon \left(\lambda \right)=\frac{{\left\|Y_{K-R}\left(\lambda \right)-BY_{R}\left(\lambda \right)\right\|}_{F}}{{\left\|Y_{K-R}\left(\lambda \right)\right\|}_{F}}$$

where **Y***_K − R_(λ*), and **Y***_R_*(*λ*) are the sensing measurements of the *λ*^th^ day of the idle participants and active participants respectively, and **B** is the spatial correlation matrix estimated based on {**Y**(*λ*); *λ* = 1, 2, and 3}. We use 11-fold cross validation to examine the approximation errors of the 11 days’ worth of Intel-Berkeley Lab sensing data.

**Figure 6:**
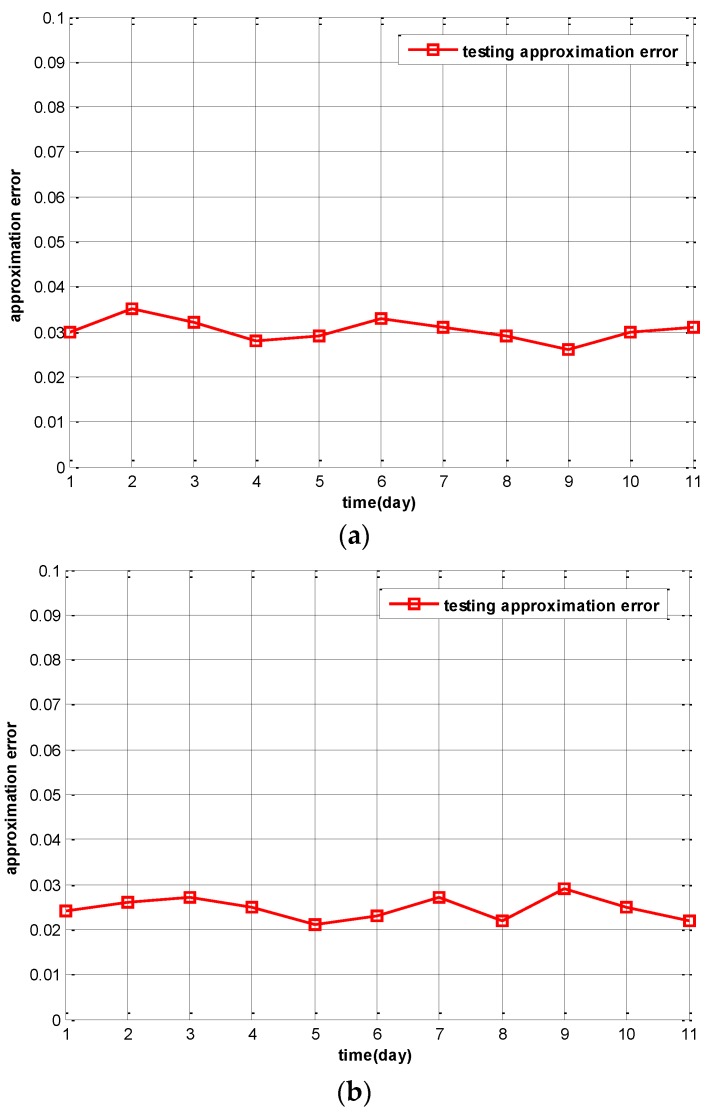

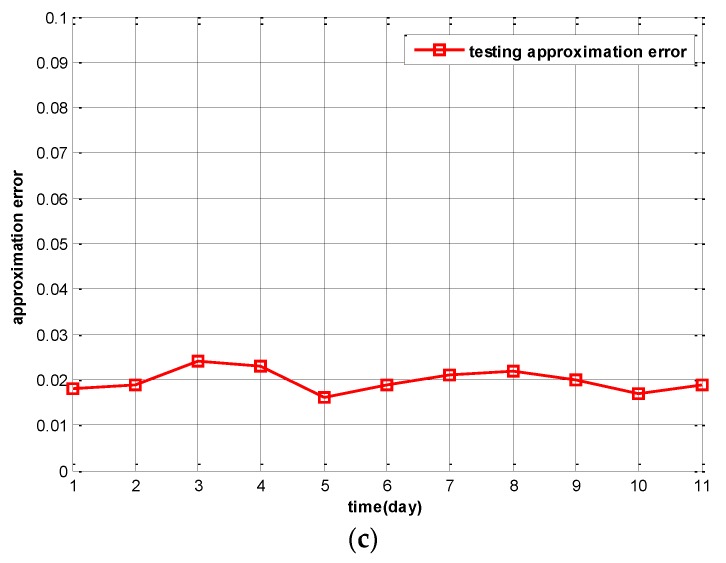
Testing approximation errors of IPSA. (**a**) 10 active participants (training loss threshold = 0.025); (**b**) 15 active participants (training loss threshold = 0.015); (**c**) 20 active participants (testing loss threshold = 0.005).

In Figure 6a, we set the training loss threshold as 0.025. It yields 10 active participants according to the IPSA algorithm. In Figure 6b, a different loss threshold of 0.015 is used and 15 participants will be active. In Figure 6c, the training loss threshold is set to 0.005 and 20 participants will be active. From the results in these figures, it is clear that the testing approximation error on different day is very stable. When more participants are active and the training loss threshold is set lower, the testing approximation error is lower. Thus, the 11-fold cross validation of Intel-Berkeley Lab sensing data proves that for a given approximation error we can adjust approximation error to approach it by selecting the appropriate number of active participants and training loss threshold.

### 5.3. Performance Comparison of IPSA, RS, and LDS

We compare the performance of IPSA against two typical geographical coverage-based participant selection schemes: the randomized selection (RS) scheme and the linear distance-based selection (LDS) scheme.

The RS algorithm intends to randomly select nodes in the sensing field. To implement the RS, we randomly select certain grids and a corresponding participant for each selected grid and put them into active mode. We then compute the corresponding approximation errors with different numbers of active grids.

LDS algorithm intends to select nodes according to their relative linear distance to central node in the clusters in the sensing field [1]. To implement the LDS algorithm, we select participants to be put into idle mode according to a probability:

(19) $$p\left(x\right)=\;\{\begin{array}{c} \gamma x/R_{center}\;\;\;\;\;\;\;\;\;\;\;\;\;\;\;\;\;\;\;\;\;\;\;0\leq x\leq x_{s};\\ 1\;\;\;\;\;\;\;\;\;\;\;\;\;\;\;\;\;\;\;\;\;\;\;\;\;\;\;\;\;\;\;\;x_{s}<x\;\leq \;R_{center} \end{array}$$

where *x* is the distance from the participants to the central participant which is located in the center of Intel Berkeley Research lab (*i.e.*, *#*4 participant) *R_center_* is the transmission radius of the central participant, and *x_s_* is a threshold determined by *R_center_* and λ, the target fraction of participants to be put into idle mode, and *γ* is a constant to enforce λ fraction of participants to be of idle mode. In this experiment, we select *R_center_* = 100, *γ* = 0.6 and *x_s_* = 100. In the Intel Berkeley Research Lab sensing field, as the length and width are 41 m and 32 m, respectively, which are smaller than the sensing field of a single cluster, the LDS in this paper is used to investigate the node-selecting strategy according to their relative linear distance to central node in whole sensing field in effect.

**Figure 7:**
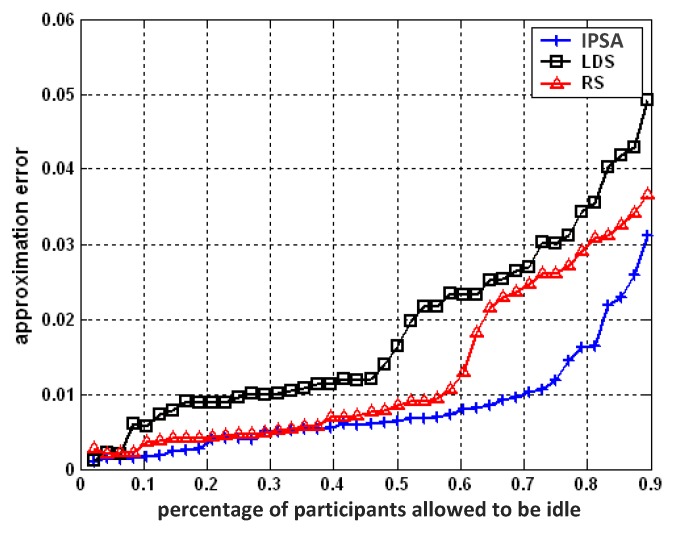
Approximation errors comparison of IPSA *vs.* RS, LDS.

For comparison purposes, we do not set a loss-threshold when running the IPSA algorithm. Instead, we tried different numbers of active participants, and plot the results as different percentages of participants in idle mode of all participants. Only lumped approximation errors corresponding to the testing data set are reported. The results are summarized in Figure 7.

It is obvious that IPSA demonstrates better performance than LDS and RS. Given the fraction of participants selected to idle mode *λ*, IPSA causes less approximation error than LDS and RS, indicating better sensing data quality. Larger *λ* will produce greater performance differences between them. From Figure 7, we can also observe that RS performs better than LDS does. According to [7], the sensing coverage degree of the LDS scheme is slightly smaller than that of the RS scheme. This could be due to the principle of LDS: allowing participants that are far away from the central participant turn to idle mode with higher probability. Border areas are not likely to be covered with active participants as a result. Less coverage degree would probably result in worse data quality.

To obtain the same information integrity, IPSA could select more participants and put them in idle mode, thus saving more energy. Referring to Figure 7, with the constraint that the approximation error is kept below 2.5% (0.025), the IPSA method needs fewer than 30% of participants (15 participants in this case) than would be required by the RS (22 participants) or LDS (29 participants).

**Figure 8:**
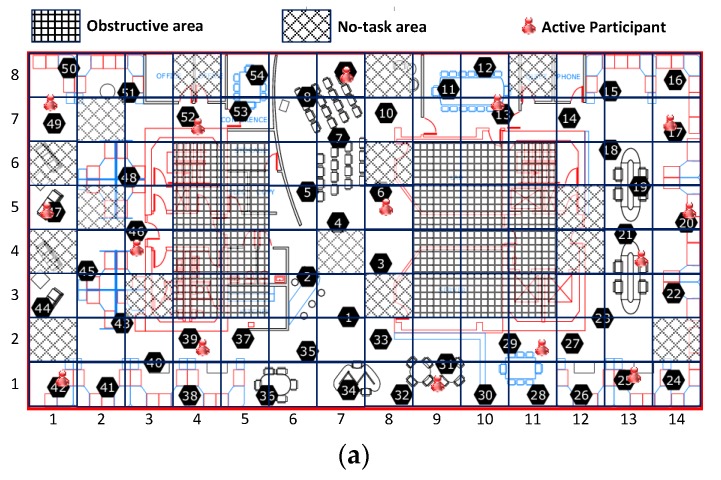

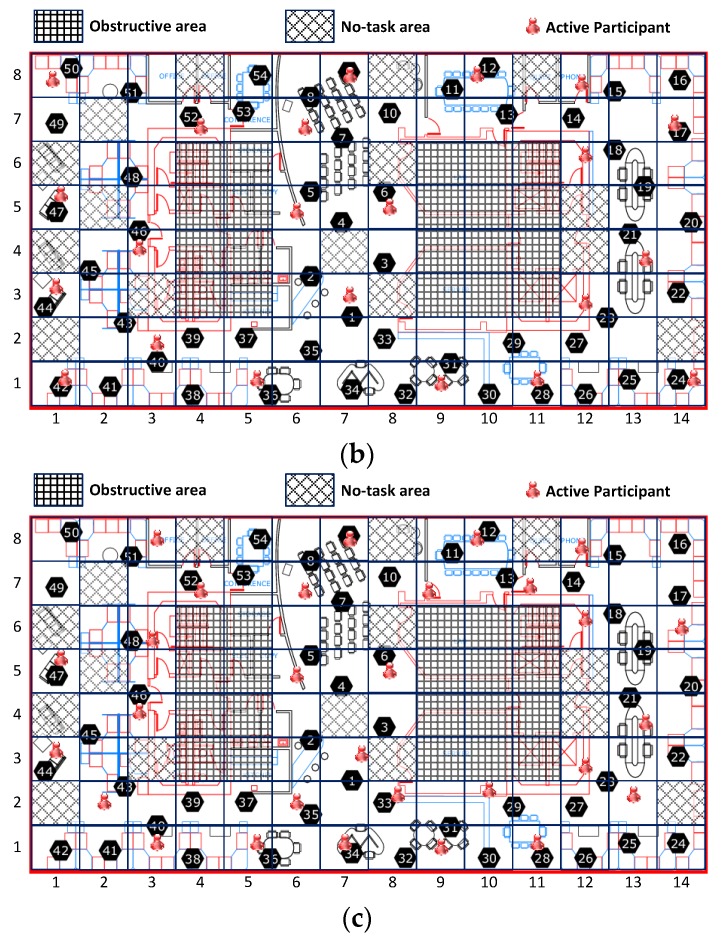
Active participants’ distribution by IPSA, RS, and LDS. (**a**) IPSA; (**b**) RS; (**c**) LDS.

Figure 8 shows the distribution of active participants by these three selection schemes. The results shown in Figure 8a further illustrated the in-homogeneous spatial correlations among sensing data as the selected active participants are cluttered at the top of the sensing field.

## 6. Conclusions

Resource consumption efficiency is one of the main problems in mobile crowd sensing systems because smart devices are limited in power, storage and data flow. One valid way to save resources is to select only a portion of the users as active participants, while keeping adequate sensing information integrity. In this paper, we proposed a novel task-centric participant selection scheme under the constraint of sensing data quality. Users are selected to participate as long as the remaining ones are able to predict their data within a given approximation error threshold. Considering the problem of resource consumption imbalance of participant selection and further cutting down the number of active participants, we explore an improved participant selection scheme. It solves the problem of selecting the minimum number of participants with some inaccurate data. Meanwhile, the fairness of selection and resource conservation can be achieved while maintaining adequate sensing data quality. Experimental results validate the effectiveness of both our task-centric cooperative sensing schemes and indicate their performance advantages.
